# Empirical and Chemical Theories

**Published:** 1893-10-21

**Authors:** Benjamin Ward Richardson


### Empirical and Chemical Therapeutics.

By Sir Benjamin Ward Richardson, M.D., F.R.S.

I endeavoured many years ago to institute a method of studying the action of remedies on the body by observing the effects of different elementary forms of remedies, that is to say, by trying how one chemical substance could be modified in its action by adding to it or abstracting from it some particular element. I designated this the method of research by the study of chemical constitution. There seemed to be something sound in this idea, conveying hopes of a progressive step on some scientific ground, and to a certain extent it has made its way. Acting on the same idea, I also endeavoured to explore in another direction. I tried to ascertain what was the precise action on living bodies of particular elements pure and simple; I tested the effects of various elements, of oxygen, hydrogen, nitrogen, chlorine, bromine, iodine, sulphur, bismuth, and others, under the impression that if we could find a specific medicinal property in the elements we could, by putting two and two together, to use a common phrase, get at fixed principles of a therapeutical order. Some very curious results emanated from this line of research, and although I did not arrive, and although no one else has arrived at such positive results as were anticipated, the labour was not thrown away.

In one sense the method appeared to be far superior to the old empirical line of inquiry which for long ages has held sway, that line which all men fifty years ago were taught, which about that time had become to some extent experimental, and which in the hands of Whytt, Wilson Philip, Broughton, Brodie, and especially Pareira, had entered on a scientific course. In its newest and broadest form the empirical, as distinct from the strict chemical mode, was based on something like the following hypothesis. Different substances, called generally medicinal substances, produce, when administered to the living body, certain different effects, produce certain different symptoms, produce, if one might venture to put it in that manner, certain distinct and different diseases. In practice therefore, we use these substances to combat diseases; we set one against the other, and either try to make the remedy combat the disease, or, it may be said, increasing the disease by the remedy, try to cure by removing the cause. As far back as 1731-4 one of the most laborious of physicians of Halle, Michael Albertus, described these two methods, one in his "Dissertatio de Curatione per Contraria," the other in his "De Curatione per Similia." In illustration, opium, which produces symptoms of stupefaction and of astringency, may be used to combat painful diarrhœa, that is *per contraria.* But painful diarrhœa may be caused by the presence of foreign substances in the alimentary canal, which the provoked purging may not be sufficient to remove. Then a dose of castor oil or other body which would promote diarrhœa may be successfully administered to enforce the removal of the foreign substance, that is *per similia*, and that is the cure.

On these lines an empirical therapeutic proceeded up to the time of what may be called the chemical era, and so it proceeds—largely—in our own day. We have found, or think we have found, medicines which reduce the animal temperature when they are administered, and thereupon we prescribe them in fevers to bring the high temperature to the natural. This is all *per contraria*, is strictly empirical from, and must never be confused with, the purely chemical process which aims to rectify by virtue of the chemical constitution of the remedies employed, and to cure by special modification of physiological function, proceeding neither on the *per contraria* nor *per similia* hypothesis, but striving to establish a series of combinations and reactions which shall govern morbid function, and destroy cause.

Suppose it were possible that we could govern function in such a manner that we could at pleasure increase or promote, or delay or arrest function, we should at once be master of every modification accidentally induced by external influences, and might be able to check on the spot all the variations which in their manifestations constitute disease. We should systematically meet the cause of the aberration; we should neutralise the effects of aberration; and by giving time for the removal of the cause, we should practically cure by a direct and immediate design. If, for example, a patient were suffering disease due to the existence in the body of some alkaloidal substance the presence of which was detectable and the action of which was readable by the symptoms, we would have it in our power, were the chemical practice perfect, to do two things—rectify at once the functional disturbance that was in progress, and by a still more advanced method change the molecular constitution of the substance that was at work producing the morbid phenomena. That would be medicine in perfection, science as true and reliable as physical transmutations carried on with organic or inorganic materials in the laboratory or workshop. When this thought was, in the first instance, in my mind, my enthusiasm led me to suggest that it was not in all cases necessary to secure absorption into the blood of remedial chemical substances, but that their action might be obtained by direct contact with the sensitive nervous surface. I took an animal recently dead from inhalation of methylene bichloride. I tied firmly all the vessels leading to and from the heart, so as to cut off all possibility of circulation of blood into or out of the heart. The heart was left with still some slight evidence of pulsation, and with clear evidence of remaining irritability on irritation. I now proceeded to instil five minims of amyl nitraite into the brain substance through the hypodermic needle, with the effect, after a momentary pause, of reaction in the motion of the heart, so that the organ recommenced to pulsate in all its parts with the same rapidity as is observed after inhalation of the nitrite by a living animal. From this observation I argued that certain foreign substances in the nervous system might be a cause of cardiac symptoms of perturbation, and that other counteracting substances used as remedies might be employed to correct the morbid functions, or to neutralise or destroy the cause of the abnormal manifestations. Chemical therapeutics brought into a science, all the steps of which being safely laid, would, I suggested, bring medicine into the pale of the exact sciences. How far that suggestion has been correct and incorrect will be seen later on in the course of these labours.

